# Current clinical immunotherapeutic approaches for head and neck cancer

**DOI:** 10.12688/f1000research.7762.1

**Published:** 2016-05-05

**Authors:** Carolina Soto Chervin, Bruce Brockstein

**Affiliations:** 1 Department of Medicine, NorthShore University HealthSystem, Evanston, Ilinois, 60201, USA; 2 Department of Medicine, University of Chicago Pritzker School of Medicine, Chicago, Illinois, USA

**Keywords:** Autologous Cell Therapy, tumor-infiltrating lymphocytes, Immune Checkpoint Inhibitors, DNA-based vaccine, Chimeric antigen receptors

## Abstract

It was estimated that 59,340 new cases of head and neck cancer would be diagnosed in the US alone in 2015 and that 12,290 deaths would be attributed to the disease. Local and regional recurrences may be treated with chemotherapy and radiation; however, metastatic head and neck cancer is fatal and is treated with chemotherapy for palliation. Recent successful treatment of a variety of solid and hematological malignancies by immunotherapeutic approaches (i.e. harnessing the body’s own immune system to combat disease) has added a fourth therapeutic option for the treatment of cancer. This commentary will review the status of immunotherapies in clinical development for the specific treatment of head and neck cancer.

## Introduction

Squamous cell cancers of the head and neck, including the oral cavity, pharynx, and larynx, together account for the eighth most common malignancy in the US and represent an even higher percentage worldwide. In 2015, a total of 59,340 new cases and 12,290 estimated deaths were expected ^1^. Despite a decrease in the number of smoking-related cases of head and neck squamous cell cancers (HNSCC), the overall incidence is steady or increased because of the epidemic of squamous cell cancers of the oropharynx attributable to human papillomavirus (HPV).

Although advances in local therapies have led to improved survival for locoregionally advanced HNSCC, fatal local recurrence and metastases remain a very significant problem. Only small advances have been made in survival rates or survival duration with various chemotherapies in the last several decades. Even with modern multi-agent chemotherapy, the expected median survival for a patient with an incurable or metastatic relapse remains under a year, or marginally longer for patients who develop metastases from an HPV-related HNSCC ^2, 3^. Improvements in systemic therapy are needed for this group of otherwise incurable patients. In addition, as systemic therapy plays a key role in the multi-modality curative intent therapy for locoregionally advanced HNSCC, better systemic therapy is needed to increase cure rates in patients with locoregionally advanced but not metastatic HNSCC.

Immunotherapy has been tested in the past in HNSCC. Trials with systemic interferon-alpha (IFN-α) or interleukin-2 (IL-2) were disappointing, and response rates ranged from 0 to 6%. One trial examining IFN-α in combination with IL-2 showed only a modest effect; two of 11 patients (18%) exhibited a partial response ^4^. Given the relative lack of success of these trials and others, and the significant toxicity of these drugs, new therapeutic approaches were needed ^4– 10^.

Standard-of-care cancer treatment has revolved around three traditional therapies: radiation, chemotherapy, and surgery. With the advent of immunotherapy, the promise of harnessing and focusing the body’s own immune system to combat malignancies has added a powerful weapon to the oncologist’s arsenal. The remainder of this review will focus on currently approved immunotherapy as well as promising immunotherapeutic approaches under clinical investigation.

## Vaccines

One of the most heavily investigated areas of cancer immunotherapy has centered on vaccines. They have been applied in most cancer indications and their lack of success has been well documented. However, there continue to be trials with new adjuvants combined with different vaccination components (e.g., peptide, DNA, and bacteria). Within head and neck cancer, a DNA-based vaccine targeting E6 and E7 genes of HPV in combination with IL-12 is currently under investigation in a phase 1 clinical trial (NCT02163057). Recent work with immunomodulatory peptide vaccines for HPV-positive and MAGE-A3-positive tumors elicited antigen-specific T cell and antibody responses to the respective vaccines but ultimately lacked clinical efficacy (NCT00257738) ^11^. Another study incorporated personalized medicine into vaccine therapy in a phase I/II trial investigating the safety profile of a vaccine composed of patient-specific tumor antigen derived from chaperone-rich tumor cell lysate (NCT01998542). A phase I trial was recently completed by using a dendritic cell p53 vaccine, but further studies need to be conducted in order to gauge clinical efficacy ^12^.

## Autologous cell therapy: tumor infiltrating lymphocytes

Adoptive cell therapy involves the transfer of tumor-reactive T cells, cultured *ex vivo*, into patients. With improvement in cell culturing (e.g., IL-2 conditioning), these T cells, which traditionally have been isolated from tumors (tumor-infiltrating lymphocytes, or TILs), could be cultured *ex vivo*, generating a sufficient number of cells before re-introduction into the patient. This therapeutic regimen was pioneered in the field of melanoma and is still employed experimentally for the treatment of various solid tumors, including HNSCC ^13, 14^. For HPV-associated HNSCC, a phase 2 clinical trial under way at the National Cancer Institute is generating TILs from metastatic HPV16/HPV18-positive tumors (NCT01585428). Undifferentiated nasopharyngeal cancers have a high association with Epstein-Barr virus (EBV) and can be treated by using EBV-specific T cells. Unlike TIL preparation, EBV-specific T cells can be isolated and expanded from peripheral blood mononuclear cells (PBMCs). Infusion of EBV-specific T cells showed a durable clinical response in four of six patients who had been refractory to chemotherapy and radiation ^15^. Recent results from a phase 2 trial of EBV-specific autologous T cells in combination with gemcitabine and carboplatin showed a 71% response rate (35 patients), but the 3-year survival rate was 37% ^16^. Currently, two clinical trials using autologous EBV-specific T cells are under way: a phase 2 trial using EBV-specific T cells as a monotherapy (NCT00431210) and a separate phase 3 trial using EBV-specific T cells in combination with gemcitabine and carboplatin versus gemcitabine or carboplatin alone (NCT02578641).

## Autologous cell therapy: chimeric antigen receptor T cell

In recent years, adoptive cell therapy has incorporated gene transfer techniques to endow T cells with receptors specific for tumor antigens. This allows the use of a naïve (with regard to antigen specificity) T cell population—a population isolated from patients’ peripheral blood rather than from a resected tumor. Chimeric antigen receptors (CARs) are composed of antibody variable domains specific for a cell surface antigen fused to intracellular T cell co-stimulatory and activation domains (4-1BB and CD3 are most commonly used). When introduced into T cells, these chimeric receptors allow the recognition and subsequent activation against a non-human leukocyte antigen (non-HLA) restricted tumor antigen. Recently, much fanfare has been focused on the use of CARs in hematological malignancies because of seminal work by Carl June and colleagues in chronic lymphocytic leukemia ^17– 19^. However, the question remains whether the successes in treating hematological cancers can be transferred to solid tumors owing not the least to the suppressive tumor microenvironment against T cells indicative of many solid tumor types (e.g., upregulation of inhibitory receptors on T cells) ^20^. Within HNSCC, there is currently an ongoing phase 1 trial using an ErbB-specific CAR ^21, 22^ (NCT01818323). Engineered T cells expressing the T1E28z CAR, an ErbB ligand fused to CD28 and CD3, will be delivered directly by injection into tumors of HNSCC patients with locoregional disease ^22^. Preclinical data of HNSCC CAR-T therapy suggest that this approach could have a therapeutic effect. A CAR recognizing the EBV latent membrane protein 1 (LMP1) exhibited specific cytolytic activity against LMP1-positive nasopharyngeal tumor line and reduced tumor growth in a xenograft model ^23^. A separate study of a CAR targeting chondroitin sulfate proteoglycan 4 (CSPG4) showed reduced tumor growth in a xenograft model of the HNSCC cell line PCI-30 ^24^.

## Immune checkpoint inhibitors

**Figure 1.**
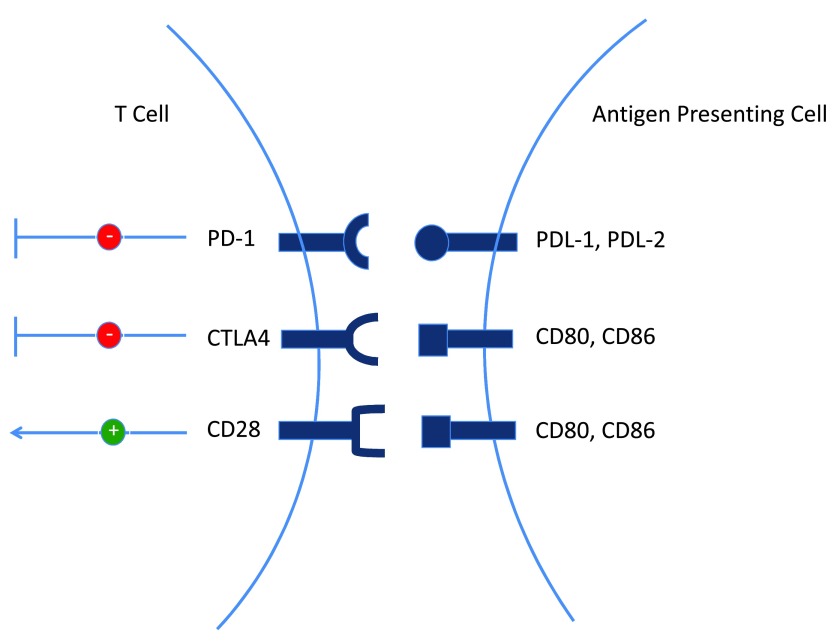
Co-stimulatory and co-inhibitory receptor-ligand interactions modulate T cell function. T cell activation is influenced by co-stimulatory signals received via CD28. Conversely, T cell activity can be reduced by upregulation of inhibitory receptors programmed death 1 (PD-1) and cytotoxic T lymphocyte-associated antigen 4 (CTLA-4).

Immunological responses are carefully regulated through calculated expression of both stimulatory and inhibitory signals. Immune checkpoint receptors provide an example of inhibitory regulation of adaptive immune responses. Their expression plays an essential role in controlling and reducing tissue damage due to robust immune responses during an infection or as a result of self-directed immunity. Programmed death 1 (PD-1) and cytotoxic T lymphocyte-associated antigen 4 (CTLA-4) are two membrane proteins expressed by T cells that have been identified as key inhibitory surface receptors in T cell function ( Figure 1). Their activation is associated with exhaustive phenotypes in tumor-infiltrating CD3 ^+^ T cells. Tumors have evolved mechanisms to successfully reduce the anti-tumor response of T cells via induction of such immune checkpoint inhibitors. As such, blockade of these receptors or their ligands is an attractive approach to promote a T cell-directed immune response against the tumor ^25^.

Nivolumab is an anti-PD-1 monoclonal antibody that was recently approved by the US Food and Drug Administration (FDA) for the treatment of advanced metastatic cancers, specifically melanoma, non-small cell lung cancer, and renal cell carcinoma. A phase 3 clinical trial demonstrated improved overall survival with nivolumab treatment compared with docetaxel chemotherapy in patients with non-small cell lung cancer (39% versus 23% at 18 months) ^26^. In patients with HNSCC refractory to platinum therapy, a phase 3 study of nivolumab monotherapy resulted in improved overall survival compared with treatment with the investigator’s choice of weekly methotrexate, docetaxel, or cetuximab (NCT02105636). Ipilimumab, an anti-CTLA-4 monoclonal antibody, was approved for the treatment of metastatic melanoma in 2011 on the basis of a phase 3 trial that compared it with a gp100 peptide vaccine ^25^. Subsequently, two PD-1 inhibitors, nivolumab and pembrolizumab, were approved for second-line use after ipilimumab. Improved progression-free survival was also observed with nivolumab compared with ipilimumab for metastatic melanoma, both as monotherapy and in combination with an additional checkpoint inhibitor, ipilimumab ^27^. Combined, ipilimumab and nivolumab extended progression-free survival by 8.5 and 4.5 months as compared with monotherapy with ipilimumab or nivolumab, respectively, and the FDA recently approved the combination for melanoma in the first-line setting for BRAF wild-type patients or following BRAF resistance. Most importantly, these drugs appear to lead to “cure” in a substantial portion of patients with metastatic melanoma, though less commonly in other cancers. The amazing success of these checkpoint inhibitors has led to a race for approval in other solid tumor indications, including HNSCC. Focusing on PD-1 blockade, or blockade of its ligand PD-L1 in advanced HNSCC, a multitude of trials are under way ( Table 1). Recent studies have concentrated on characterizing PD-1/PD-L1 expression and its correlation with overall outcome. HPV-positive HNSCC tumors were shown to have a unique immune profile compared with HPV-negative tumors. They are associated with a higher density of CD8 ^+^ T cells, which can be stimulated to produce IFN-γ and IL-17 *in vitro* ^28^. Although efforts have focused on the profile of PD-1/PD-L1 intra-tumoral expression, there is a lack of data correlating expression in HPV-positive HNSCC with overall survival ^28– 30^. In fact, at least one study unexpectedly correlated improved overall outcomes with increased levels of PD-1 ^+^ T cells in HPV-positive tumors ^31^.

**Table 1.** PD-1/PD-L1 blockade clinical trials in advanced head and neck squamous cell cancer.

Phase	Study	Agent (target)	National Clinical Trial number	Status
II	PDR001 monotherapy for nasopharyngeal carcinoma refractory to standard therapy	PDR001 (Anti-PD-1)	NCT02605967	Not yet recruiting
I/II	LAG525 monotherapy and in combination with PDR001 for advanced malignancies	PDR001 (Anti-PD-1) LAG525 (Anti-LAG-3)	NCT02460224	Recruiting
III	Nivolumab monotherapy for HNSCC refractory to platinum therapy	Nivolumab (Anti-PD-1)	NCT02105636	Active, not recruiting
I/II	Urelumab in combination with nivolumab for advanced/metastatic solid tumors and B cell non-Hodgkin’s lymphoma	Nivolumab (Anti-PD-1) Urelumab (Anti-CD137)	NCT02253992	Recruiting
II	Nivolumab and HPV-16 vaccination for HPV-16-positive incurable solid tumors	Nivolumab (Anti-PD-1)	NCT02426892	Not yet recruiting
II	Nivolumab monotherapy for recurrent and metastatic nasopharyngeal carcinoma	Nivolumab (Anti-PD-1)	NCT02339558	Recruiting
I/II	Varlilumab in combination with nivolumab for advanced refractory solid tumors	Nivolumab (Anti-PD-1) Varlilumab (Anti-CD27)	NCT02335918	Recruiting
III	MEDI4736 monotherapy or combined with tremelimumab versus standard therapy for recurrent/metastatic HNSCC	MEDI4736/Durvalumab (Anti-PD-L1) Tremelimumab (Anti-CTLA-4)	NCT02369874	Recruiting
II	MEDI4736 and tremelimumab monotherapy or combination for recurrent/ metastatic HNSCC	MEDI4736/Durvalumab (Anti-PD-L1) Tremelimumab (Anti-CTLA-4)	NCT02319044	Recruiting
I/II	MEDI4736 monotherapy for advanced solid tumors	MEDI4736/Durvalumab (Anti-PD-L1)	NCT01693562	Recruiting
II	MEDI4736 monotherapy for recurrent/ metastatic HNSCC	MEDI4736/Durvalumab (Anti-PD-L1)	NCT02207530	Recruiting
I/II	ADXS11-001 or MEDI4736 monotherapy or combination for advanced or metastatic cervical or HPV ^+^ HNSCC	MEDI4736/Durvalumab (Anti-PD-L1) ADXS11-001 (tLLO-HPV-16-E7- positive Listeria)	NCT02291055	Active, not recruiting
Ib/II	MEDI4736 in combination with AZD9150 or AZD5069 for metastatic HNSCC	MEDI4736/Durvalumab (Anti-PD-L1) AZD9150 (STAT3 antisense) AZD5069 (CXCR2 antagonist)	NCT02499328	Recruiting
I	MEDI4736 in combination with tremelimumab for recurrent or metastatic HNSCC	MEDI4736/Durvalumab (Anti-PD-L1) Tremelimumab (Anti-CTLA-4)	NCT02262741	Recruiting
III	MEDI4736 monotherapy or in combination with tremelimumab compared with standard therapy for first-line recurrent or metastatic HNSCC	MEDI4736/Durvalumab (Anti-PD-L1) Tremelimumab (Anti-CTLA-4)	NCT02551159	Recruiting
I/IIa	MK-3475 in combination with PLX3397 for advanced melanoma and other solid tumors	MK-3475/Pembrolizumab (Anti-PD-1) PLX3397 (SF1R Inhibitor)	NCT02452424	Recruiting
I	MK-3475 in combination with MGA271 for melanoma, HNSCC, and non-small-cell lung cancer	MK-3475/Pembrolizumab (Anti-PD-1) MGA271 (Anti-B7-H3)	NCT02475213	Recruiting
I/II	MK-3475 in combination with vorinostat for recurrent or metastatic HNSCC and salivary gland malignancies	MK-3475/Pembrolizumab (Anti-PD-1) Vorinostat	NCT02538510	Recruiting
II	MK-3475 monotherapy or in combination with ACP-196 for advanced HNSCC	MK-3475/Pembrolizumab (Anti-PD-1) ACP-196 (Btk inhibitor)	NCT02454179	Recruiting
Ib	MK-3475 in combination with chemo radiotherapy for locally advanced HNSCC	MK-3475/Pembrolizumab (Anti-PD-1)	NCT02586207	Recruiting
II	MK-3475 in combination with radiation for HNSCC ineligible for cisplatin	MK-3475/Pembrolizumab (Anti-PD-1)	NCT02609503	Not yet recruiting
II	MK-3475 monotherapy for locoregionally advanced, surgically resectable HNSCC	MK-3475/Pembrolizumab (Anti-PD-1)	NCT02296684	Recruiting
II	MK-3475 with reirradiation for locoregional inoperable recurrence or second primary HNSCC	MK-3475/Pembrolizumab (Anti-PD-1)	NCT02289209	Recruiting
II	MK-3475 monotherapy for recurrent or metastatic HNSCC refractory to platinum and cetuximab	MK-3475/Pembrolizumab (Anti-PD-1)	NCT02255097	Active, Not recruiting
III	MK-3475 monotherapy or in combination with chemotherapy for recurrent or metastatic HNSCC	MK-3475/Pembrolizumab (Anti-PD-1)	NCT02358031	Recruiting
III	MK-3475 monotherapy versus standard therapy (methotrexate, docetaxel, or cetuximab) for recurrent or metastatic HNSCC	MK-3475/Pembrolizumab (Anti-PD-1)	NCT02252042	Recruiting

CTLA-4, cytotoxic T lymphocyte-associated antigen 4; HNSCC, head and neck squamous cell cancer; HPV, human papillomavirus; PD-1, programmed death 1; PD-L1, programmed death-ligand 1.

## Summary

It remains to be seen whether adoptive cellular therapy alone will be sufficient to affect a sustainable anti-tumor response in HNSCC. The approvals of checkpoint inhibitor antibodies for melanoma (ipilimumab, nivolumab, and pembrolizumab), non-small cell lung carcinoma (nivolumab and pembrolizumab), and most recently renal cell carcinoma (nivolumab) have paved the way for these foundational therapies; that is, one or more checkpoint inhibitors will likely be incorporated into solid tumor therapeutic regimens. As such, there is an extensive clinical trial program for approval in new indications, including cancers of the head and neck. It is not hard to imagine that the next generation of immunotherapy would combine these checkpoint inhibitors with engineered T cells (e.g., CARs and modified T cell receptors) or even autologous T cells.

However, the attractiveness of antibody therapy is offset by the headache of cellular therapy. Whereas checkpoint inhibitors represent classic “off-the-shelf” biopharmaceutical agents, cellular therapy remains “personalized” medicine. From a manufacturing/business standpoint, it is still not yet feasible to mass-produce cellular therapies, although industry is hard at work solving this problem. As such, adoptive therapy approaches are still years away from “off-the-shelf” status ^32, 33^.
